# Discovery of Prostamide F_2α_ and Its Role in Inflammatory Pain and Dorsal Horn Nociceptive Neuron Hyperexcitability

**DOI:** 10.1371/journal.pone.0031111

**Published:** 2012-02-21

**Authors:** Luisa Gatta, Fabiana Piscitelli, Catia Giordano, Serena Boccella, Aron Lichtman, Sabatino Maione, Vincenzo Di Marzo

**Affiliations:** 1 Endocannabinoid Research Group, Department of Experimental Medicine–Division of Pharmacology “L. Donatelli”, Second University of Naples, Naples, Italy; 2 Endocannabinoid Research Group, Institute of Biomolecular Chemistry, C.N.R., Pozzuoli, Italy; 3 Department of Pharmacology and Toxicology, Virginia Commonwealth University, School of Medicine, Richmond, Virginia, United States of America; Universidad Federal de Santa Catarina, Brazil

## Abstract

It was suggested that endocannabinoids are metabolized by cyclooxygenase (COX)-2 in the spinal cord of rats with kaolin/λ-carrageenan-induced knee inflammation, and that this mechanism contributes to the analgesic effects of COX-2 inhibitors in this experimental model. We report the development of a specific method for the identification of endocannabinoid COX-2 metabolites, its application to measure the levels of these compounds in tissues, and the finding of prostamide F_2α_ (PMF_2α_) in mice with knee inflammation. Whereas the levels of spinal endocannabinoids were not significantly altered by kaolin/λ-carrageenan-induced knee inflammation, those of the COX-2 metabolite of AEA, PMF_2α_, were strongly elevated. The formation of PMF_2α_ was reduced by indomethacin (a non-selective COX inhibitor), NS-398 (a selective COX-2 inhibitor) and SC-560 (a selective COX-1 inhibitor). In healthy mice, spinal application of PMF_2α_ increased the firing of nociceptive (NS) neurons, and correspondingly reduced the threshold of paw withdrawal latency (PWL). These effects were attenuated by the PMF_2α_ receptor antagonist AGN211336, but not by the FP receptor antagonist AL8810. Also prostaglandin F_2α_ increased NS neuron firing and reduced the threshold of PWL in healthy mice, and these effects were antagonized by AL8810, and not by AGN211336. In mice with kaolin/λ-carrageenan-induced knee inflammation, AGN211336, but not AL8810, reduced the inflammation-induced NS neuron firing and reduction of PWL. These findings suggest that inflammation-induced, and prostanoid-mediated, enhancement of dorsal horn NS neuron firing stimulates the production of spinal PMF_2α_, which in turn contributes to further NS neuron firing and pain transmission by activating specific receptors.

## Introduction

Activation of cannabinoid receptors of type-1 (CB1) and/or -2 (CB2) by synthetic agonists as well as by the two most studied endocannabinoids, anandamide (AEA) and 2-arachidonoylglycerol (2-AG), has been proposed as a novel anti-hyperalgesic strategy based on studies carried out in several experimental models of inflammatory and neuropathic pain [1], [2]. In particular, selective inhibitors of endocannabinoid inactivation by the hydrolytic enzymes monoacylglycerol lipase (MAGL, specific for 2-AG) or, particularly, fatty acid amide hydrolase (FAAH, which can inactivate both AEA and 2-AG), were suggested to represent a safe and efficacious way of inhibiting pain without the central side effects that usually limit the use of the natural agonist of cannabinoid receptor, delta^9^-tetrahydrocannabinol [3], [4]. However, a recent clinical study, presented at the 2010 Conference of the International Association for the Study of Pain, showed that a selective and potent FAAH inhibitor, PF-04457845 [5], was not efficacious at reducing pain in patients with osteoarthritis of the knee [6]. This unexpected result may have several explanations, ranging from simple differences between man and rodents to the observation that inhibition of FAAH also prolongs the action of bioactive fatty amides other than AEA, which do not necessarily inhibit pain. However, a recent animal study, carried out in a model of knee inflammation, suggested that endocannabinoids, during this pathological condition, may also be inactivated by enzymes other than FAAH, and in particular by cyclooxygenase-2 (COX-2) [7]. In this previous study, the authors suggested that the anti-hyperalgesic effect of selective COX-2 inhibitors in rats with knee inflammation induced by various inflammatory stimuli, and the inhibition of the underlying hyperexcitability of dorsal horn nociceptive (NS) neurons by these compounds, was due, at least in part, to inhibition of 2-AG oxidation by COX-2, subsequent elevation of spinal 2-AG levels and indirect activation of spinal CB1 receptors [7]. Clearly, if during knee inflammation, endocannabinoids are substrates also for COX-2, inhibition of FAAH alone would not be sufficient to counteract their inactivation, and might even favor the COX-2-catalysed formation of endocannabinoid-derived oxidation products, which might exert pro-inflammatory and pro-algesic effects per se, as suggested previously [8], via specific and yet to be fully identified non-cannabinoid, non-prostanoid receptors [9]. In support of this possibility, a prostaglandin F synthase isoform with activity on the “AEA-endoperoxyde” derived from COX-2 was recently cloned and identified in myelin sheaths of the mouse brain and spinal cord [10]. However, no molecular evidence for the occurrence of prostaglandin-like derivatives of AEA has been reported to date in vivo in animals, under either physiological or pathological conditions. The only available data on the formation of AEA COX-2 derivatives in vivo is from studies in which FAAH^−/−^ mice were treated with exogenous AEA [11], and even evidence in vitro was obtained only in cells treated with either exogenous AEA [12] or, more recently, a non-physiological stimulus such as ionomycin to increase the intracellular levels of AEA [13].

In view of these considerations, and of the increasingly accepted role of COX-2 in the inactivation of endocannabinoids in both spinal [14] and supra-spinal [15], [16] structures (role that first emerged when it became clear that both AEA and 2-AG are good substrates for this enzyme in vitro [17], [18]), we have investigated here whether COX-2 metabolites of AEA and 2-AG, known as prostaglandin-ethanolamides (or prostamides [PMs]) and prostaglandin-glycerol esters (PG-GEs) are formed in the spinal cord of mice with knee inflammation, and if they play any role in NS neuron hyperexcitability and hyperalgesia. With this purpose, we developed a novel analytical technique, using liquid chromatography-ion trap-time of flight-tandem mass spectrometry (LC-IT-ToF MS-MS), for the unequivocal identification and quantification of the major PMs and PG-GEs, and tested the effects of one of these compounds, as well as of selective antagonists for its proposed receptor, on pain perception and NS neuron hyperexcitability, in healthy and/or knee-inflamed mice. We report data suggesting that PMF_2α_ is produced in the spinal cord of mice with knee inflammation and contributes to inflammatory hyperalgesia.

## Results

### An LC-IT-ToF MS-MS method for the quantification of endocannabinoid COX-2 derivatives

**Figure 1:**
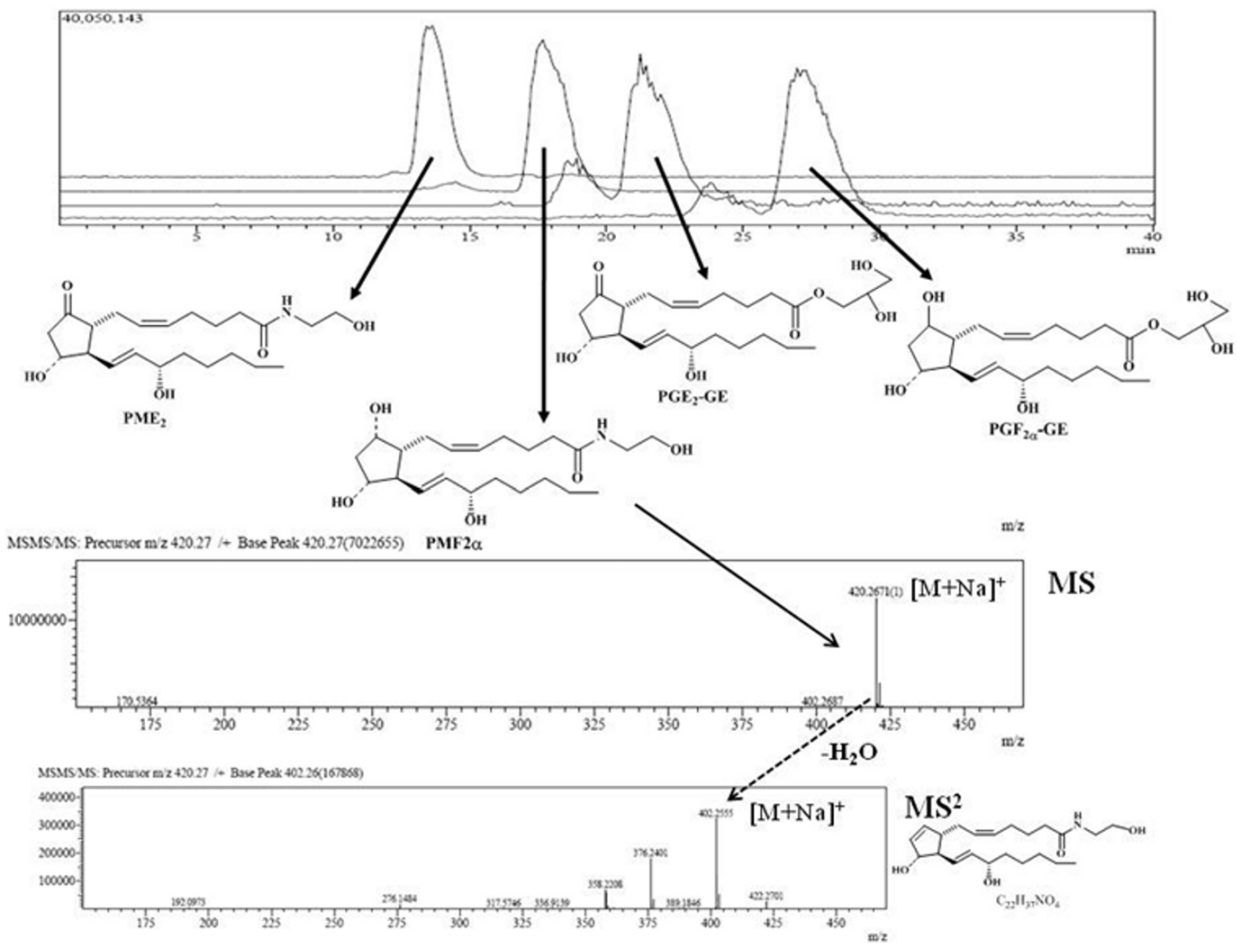
Representative extracted ion chromatogram of a pre-purified lipid extract from a rat brain homogenate spiked with synthetic standards of prostamides and prostaglandin-glycerol esters (100 pmol each). LC parameters were optimized to ensure good separation among the analytes (PME_2_, PMF_2α_, PGE_2_-GE and PGF_2α_–GE). Shown in the bottom panel are an example of positive-ion electrospray mass spectrum of a major component of this spiked homogenate, the PMF_2α_ precursor ion (m/z 420.2671), as a sodium adduct, and the corresponding product ions for the CID of the fragment with m/z 420.2671 in the MS-MS spectra (m/z 402.2555), which in turn corresponds to the C_22_H_37_NO_4_ sodiated fragment, after loss of water. Pre-purified rat brain lipid extracts do not contain measurable amount of endogenous prostamides (not shown). Instead, pre-purified mouse spinal cord extracts (not shown) only contain PMF_2α_ in measurable although much smaller amounts than those shown here (see Fig. 2).

In order to validate our method, we spiked a rat brain homogenate with synthetic standards of PME_2_, PMF_2α_, PGE_2_-GE and PGF_2α_-GE (100 pmol each). After lipid extraction and pre-purification (see Methods), the extract was analysed by the LC-IT-TOF method described here, and the four compounds exhibited retention times of 13.5, 18.5, 22.5 and 29 min, respectively (Fig. 1). Prostamides and prostaglandin-GE quantification was performed by isotope dilution by using *m/z* values of 422.2815 and 418.2564 corresponding to the sodium adduct of the molecular ion [M+23]^+^ for deuterated and undeuterated PME_2_, respectively; or *m/z* values of 424.2972 and 420.2726 corresponding to the sodium adduct of the molecular ion [M+23]^+^ for deuterated and undeuterated PMF_2α_; or *m/z* values of 453.2761 and 449.2515 corresponding to the sodium adduct of the molecular ion [M+23]^+^ for deuterated and undeuterated PGE_2_-GE; and *m/z* values of 455.2917 and 451.2923 for PMF_2α_-GE (Fig. 1). The full recovery of PME_2_, PMF_2α_, PGE_2_-GE and PGF_2α_-GE from tissue due to the analytical procedure reported above was 42.5±1.9, 61.6±15.9, 49.1±15.7 and 52.3±17.8%, respectively.

As assessed by using pure standards, the LC-ESI-IT-ToF method described here for the first time is specific and sensitive with a limit of detection (defined as the concentration at which the signal/noise ratio is greater than 3∶1) of 25 fmol in the MS mode and 500 fmol in the MS-MS mode for all the compounds analysed. Moreover, the ratio between the [M+23]^+^ peak areas of pure undeuterated (0.05–20 pmol) *vs.* pure deuterated (1 pmol) PME_2_, PMF_2α_, PGE_2_-GE and PGF_2α_-GE varied linearly with the amount of the respective undeuterated standards. The quantification limit of compounds was 50 fmol and the reproducibility of the method ranged between 95% and 99%.

### Induction of knee inflammation is accompanied by elevation of PMF_2α_ levels in the spinal cord

**Figure 2:**
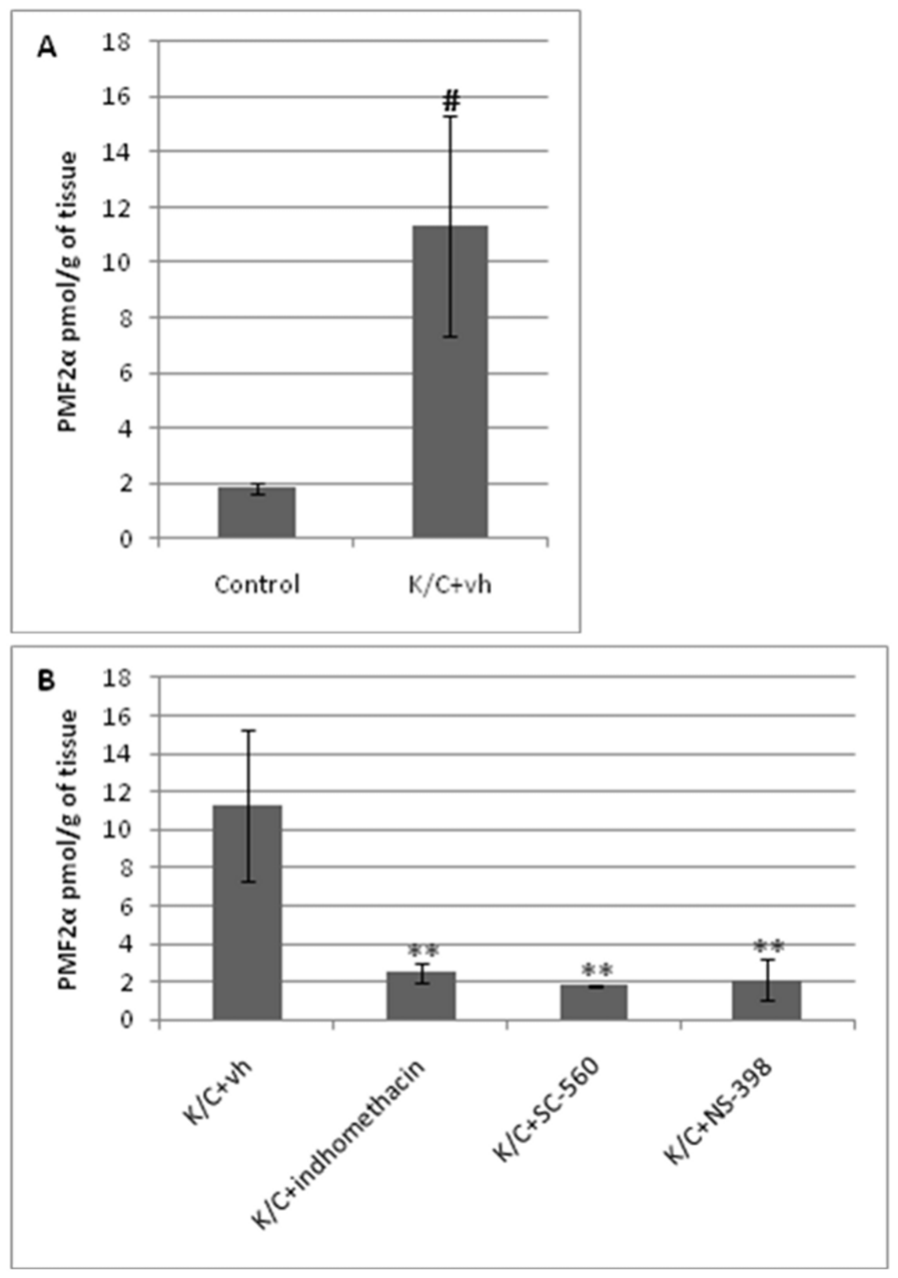
Levels of PMF_2α_ in the spinal cord after the induction of inflammation with kaolin/λ-carrageenan (K/C) (A) and after the administration of COX inhibitors (B). Data are means ± SEM of separate determinations in N = 5 rats. ^#^ P<0.05 Control *vs.* K/C+vh; ** P<0.01 K/C+vh vs. K/C+indomethacin-K/C+SC560-K/C+NS398.

The levels of PMF_2α_ increased significantly (P<0.05; Fig. 2A) after the induction of inflammation caused by the administration of the solution of kaolin-λ-carrageenan. The other COX-2 derivatives of AEA and 2-AG were below detection limit. Considering that ∼50 mg of wet tissue were extracted and analysed, and bearing in mind the yield of the extraction and purification procedures (see above), we can estimate that in the spinal cord of inflamed knee mice the amount of PME_2_, PGE_2_-GE and PGF_2α_-GE is less than ∼1.2, 1.0 and 1.0 pmol/g wet tissue weight, respectively. Finally, the spinal levels of AEA and 2-AG were not significantly altered by any of the treatments (Table 1).

**Table 1:** Concentrations of anandamide (AEA) and 2-arachidonoylglycerol in the spinal cord of healthy and inflamed knee mice, and after treatment with COX inhibitors.

	AEA(pmol/g of tissue)	2-AG(pmol/mg of tissue)
**Control**	55.04±4.03	23.66±1.72
**K/C+vehicle**	43.55±6.17	26.43±1.35
**K/C+indomethacin**	51.03±3.94	28.85±1.54
**K/C+SC-560**	51.67±3.11	27.81±4.27
**K/C+NS398**	48.76±8.52	26.50±3.34

Data are means ± SEMs of separate determinations in N = 5 mice. No statistically significant difference between groups was found (as assessed by ANOVA followed by Bonferroni's post-hoc test). K/C, kaolin/λ-carrageenan.

### Inhibition of COX-1 and COX-2 counteracts knee inflammation-induced elevation of PMF_2α_ levels

As shown in Figure 2B, the inhibition of COX-1 or COX-2 or both significantly decreased (P<0.01) PMF_2α_ levels in the spinal cord. In particular, the non-selective COX inhibitor, indomethacin, and the selective COX-1 inhibitor, SC-560, produced the same effect as NS-398 (a selective COX-2 inhibitor).

### Effect of PMF_2α_ alone or in combination with AL8810 or AGN211336 on the spontaneous activity of NS neurons in healthy rats

**Figure 3:**
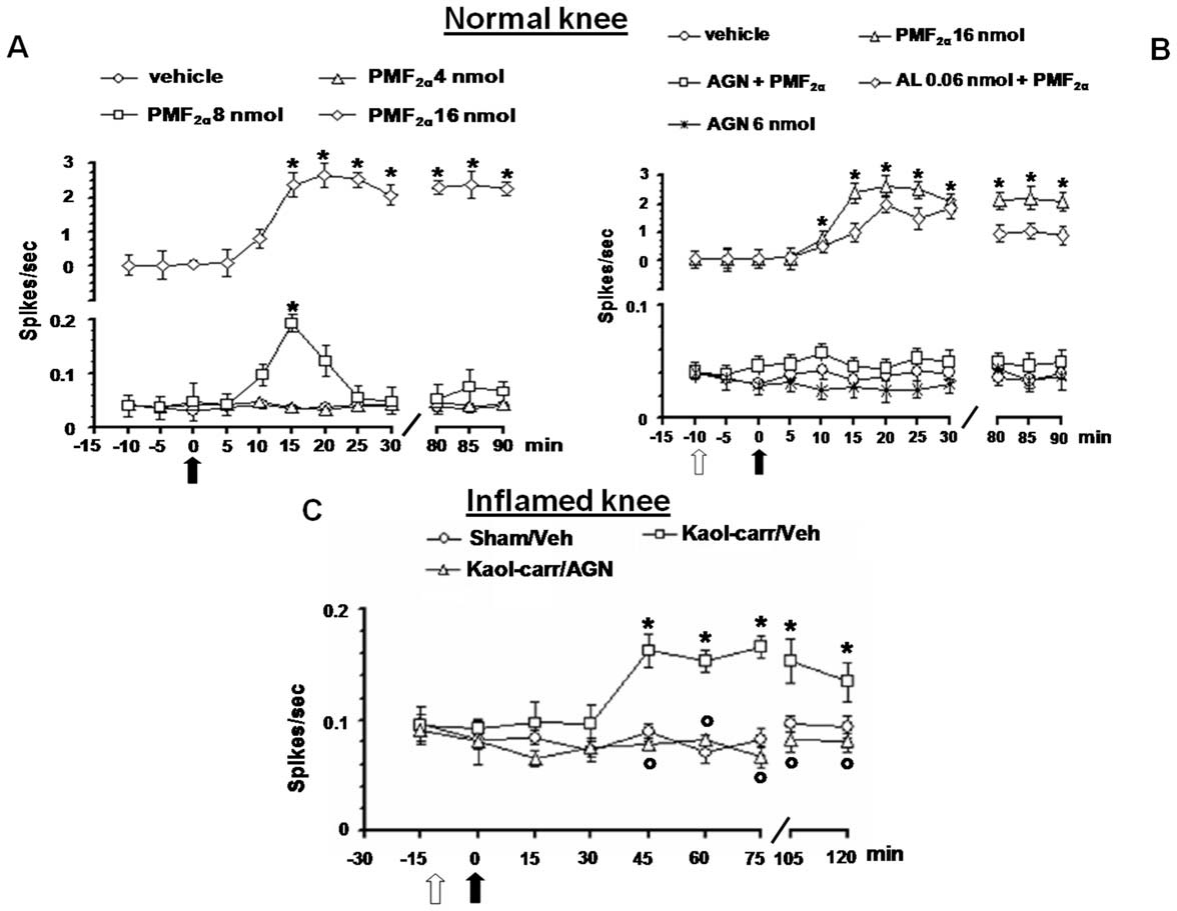
Effects of spinal application (microinjections) of vehicle (0.05% DMSO in ACSF), PMF_2α_ (4, 8 and 16 nmol) alone (A), or in combination with AGN 211336 (6 nmol) or AL 8810 (0.06 nmol) (B), on the spontaneous firing of NS neurons in normal knee mice. Effects of spinal application of vehicle (0.05% DMSO in ACSF) and AGN 211336 (6 nmol) on the spontaneous firing of NS neurons in normal knee (B), in sham and in inflamed knee mice (C). Vehicle or drugs were administered at time 0 whereas AGN 211336 (6 nmol) or AL 8810 (0.06 nmol) were administered 10 min before. Black arrow indicates vehicle or agonist spinal application while white arrow indicates antagonist spinal injection. Each point represents the mean ± S.E.M of 6–7 neurons of different treated group of mice. * indicates statistically significant difference versus vehicle (A and B) or versus sham/veh (C), and ° versus PMF_2α_ (16 nmol) (B) or versus kaolin/λ-carrageenan (C). P values<0.05 were considered statistically significant (one-way ANOVA).

The results are based on recordings from spinal cord NS neurons (one cell recorded from each animal per treatment) at a depth of 700–1000 µm from the surface of the spinal cord. This cell population was characterized by a mean rate of spontaneous firing of 0.04±0.02 spikes/sec, and only cells which showed this mean firing were chosen to measure post-drug changes in their spontaneous activity. Topical spinal cord application of vehicle (0.05% DMSO in artificial cerebrospinal fluid [ACSF]) did not change the spontaneous activity of NS neurons (0.033±0.003 spikes/sec) (Fig. 3A and 3B). Microinjections of PMF_2α_ (8 and 16 nmol) significantly increased NS cell activity in a dose-dependent manner (0.12±0.02 and 2.65±0.35 spikes/sec, respectively; *P*<0.05) (Fig. 3A). The lower dose (4 nmol) of PMF_2α_ did not change NS spontaneous activity. Pre-treatment with AL8810 (0.06 nmol) did not prevent the effect of PMF_2α_ (16 nmol) on NS cell spontaneous activity (Fig. 3B), which was instead completely abolished by 10 min pre-treatment with AGN211336 (6 nmol). AGN211336 (6 nmol) was inactive per se (Fig. 3B).

### Effect of AGN211336 on the spontaneous activity of NS neurons in inflamed knee rats

The firing of NS neurons was also examined in sham and inflamed knee rats. The induction of inflammation in the mouse knee caused a significant increase in NS spontaneous activity compared to sham/veh group (0.16±0.015 spikes/sec; *P*<0.05). This effect was observed 45 min after knee joint injection of kaolin and λ-carrageenan and remained at this level for the duration of recording period (120 min) (Fig. 3C). Topical spinal cord application of vehicle (0.05% DMSO in ACSF) did not change the increased spontaneous activity of NS neurons induced by inflammation (0.084±0.006 spikes/sec) (Fig. 3C), whereas pre-treatment with AGN211336 (6 nmol) completely antagonized the effect of inflammation on NS cell spontaneous activity (0.078±0.005 spikes/sec) (Fig. 3C).

### Effect of PMF_2α_ alone or in combination with AL8810 or AGN211336 on the evoked activity of NS neurons in healthy rats

**Figure 4:**
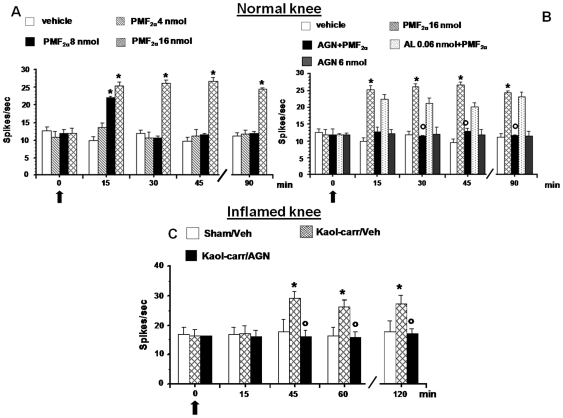
Effects of spinal application (microinjections) of vehicle (0.05% DMSO in ACSF), PMF_2α_ (4, 8 and 16 nmol) alone (A) or in combination with AGN 211336 (6 nmol) or AL 8810 (0.06 nmol) (B) on evoked activity of NS neurons in normal knee mice. Effects of spinal application of vehicle (0.05% DMSO in ACSF) and AGN 211336 (6 nmol) on evoked activity of NS neurons in normal knee (B), in sham and in inflamed knee mice (C). Vehicle or drugs were administered at time 0, as indicated by the black arrow, whereas AGN 211336 (6 nmol) or AL 8810 (0.06 nmol) were administered 10 min before (not shown). Each point represents the mean ± S.E.M of 6–7 neurons of different groups of mice. * indicates statistically significant difference versus vehicle (A and B) or versus sham/veh (C), and ° versus PMF_2α_ (16 nmol) (B) or versus kaolin/λ-carrageenan (C). P values<0.05 were considered statistically significant (one-way ANOVA).

The evoked activity of NS neurons was also examined following paw mechanical stimulation by von Frey filament with bending force of 97.8 mN for 2 s. Topical spinal cord application of vehicle (0.05% DMSO in ACSF) did not change the evoked activity of NS neurons (10±0.1 spikes/sec) (Fig. 4A and 4B). Microinjections of PMF_2α_ (8 and 16 nmol) significantly increased the NS cell evoked activity in a dose dependent manner (22±0.47 and 25.3±1.13 spikes/sec, respectively; *P*<0.05), (Fig. 4A). The lower dose (4 nmol) of PMF_2α_ did not change NS evoked activity. Pre-treatment with AL8810 (0.06 nmol) did not prevent the effect of PMF_2α_ (16 nmol) on NS cell evoked activity (Fig. 4B), which was instead completely abolished by 10 min pre-treatment with AGN211336 (6 nmol). AGN211336 (6 nmol) was inactive per se (Fig. 4B).

### Effect of AGN 211336 on the evoked activity of NS neurons in inflamed knee rats

The evoked activity of NS neurons was also examined in sham and inflamed knee rats. The induction of inflammation in the mouse knee caused a significant increase in NS evoked activity compared to the sham/veh group (16.9±2.5 spikes/sec); *P*<0.05). This effect was observed 45 min after injection and remained at this level for the duration of the recording period (120 min) (Fig. 4C). Topical spinal cord application of vehicle (0.05% DMSO in ACSF) did not change the increased spontaneous activity of NS neurons induced by inflammation (29±2.37 spikes/sec (Fig. 4C), whereas pre-treatment with AGN 211336 (6 nmol) completely antagonized the effect of inflammation on NS cell evoked activity (16.29±2.16 spikes/sec) (Fig. 4C).

### Effect of PMF_2α_ alone or in combination with AL8810 or AGN211336 on PWL in healthy rats

**Figure 5:**
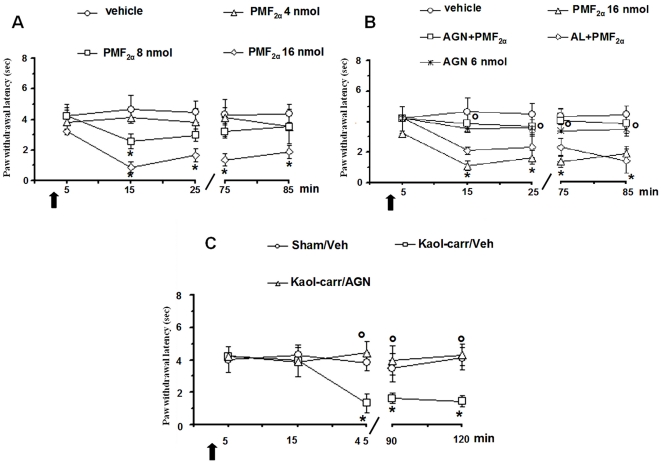
Effects of spinal application (microinjections) of vehicle (0.05% DMSO in ACSF), PMF_2α_ (4, 8 and 16 nmol) alone (A) or in combination with AGN 211336 (6 nmol) or AL 8810 (0.06 nmol) (B) on paw withdrawal thresholds (PWT) in normal knee mice. Effects of spinal application of vehicle (0.05% DMSO in ACSF) and AGN 211336 (6 nmol) on paw withdrawal latency (PWL) in normal knee (B), in sham and in inflamed knee mice (C). Vehicle or drugs were administered at time 0, as indicated by the black arrow, whereas AGN 211336 (6 nmol) or AL 8810 (0.06 nmol) were administered 10 min before (not shown). Each point represents the mean ± S.E.M of 6–7 neurons of different treated group of mice. * indicates statistically significant difference versus vehicle (A and B) or versus sham/veh (C), and ° versus PMF_2α_ (16 nmol) (B) or versus kaolin/λ-carrageenan (C). P values<0.05 were considered statistically significant (one-way ANOVA).

Topical spinal cord application of vehicle (0.05% DMSO in ACSF) did not change the PWL (4.2±0.3 sec) (Fig. 5A and 5B). Microinjections of PMF_2α_ (8 and 16 nmol) significantly reduced the PWL in a dose-dependent manner (2.5±0.5 and 1.35±0.37 sec, respectively; *P*<0.05) (Fig. 5A). The lower dose (4 nmol) of PMF_2α_ did not change PWL. Pre-treatment with AL8810 (0.06 nmol) did not prevent the effect of PMF_2α_ (16 nmol) (Fig. 5B), which was instead completely abolished by 10 min pre-treatment with AGN 211336 (6 nmol). AGN 211336 (6 nmol) was inactive per se (Fig. 5B).

### Effect of AGN 211336 on PWL in inflamed knee rats

The PWL was also examined in sham and inflamed knee rats. The induction of inflammation in the mouse knee caused a significant reduction in PWL compared to sham/veh group (4±0.8 sec; *P*<0.05). This effect was observed 45 min after injection and remained at this level for the duration of all the period of observation (120 min) (Fig. 5C). Topical spinal cord application of vehicle (0.05% DMSO in ACSF) did not change the reduced PWL caused by inflammation (1.52±0.2 sec) (Fig. 5C), whereas pre-treatment with AGN 211336 (6 nmol) completely antagonized the effects of inflammation on mouse PWL (3.9±0.9 sec) (Fig. 5C).

**Figure 6:**
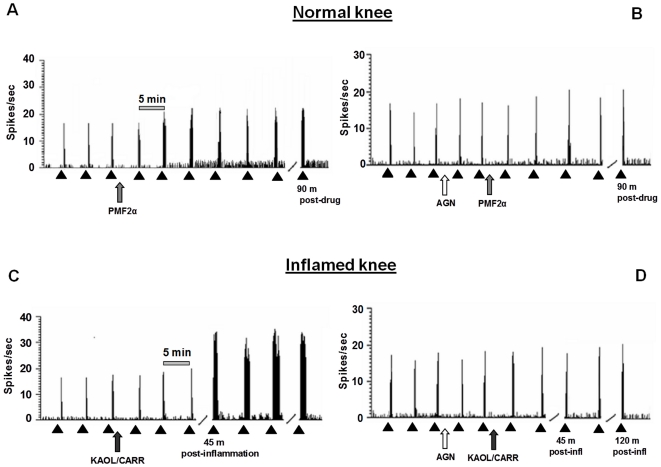
Representative ratemeters showing the spontaneous activity and the responses to a noxious stimulation (von Frey filaments 97.8 mN/2 sec) of a single NS neuron both before and after spinal application of PMF_2α_ (16 nmol) alone, which increased the spontaneous activity and the evoked activity of NS neurons (A), or in combination with AGN 211336 (6 nmol) which did not alter either NS spontaneous activity or the noxious stimulation-evoked activity in normal knee mice (B). PMF_2α_ (16 nmol) was also administered both before and after knee joint injection of kaolin/λ-carrageenan, which alone increased the spontaneous activity and the noxious stimulation evoked activity of NS neurons (C), or in combination with AGN 211336 (6 nmol), which prevented the effect induced by inflammation on NS spontaneous activity or the noxious stimulation-evoked activity in inflamed knee mice (D). Scale grey bar indicates 5 min intervals for ratemeter records and small black arrows indicate the noxious stimulation on mouse hind paw.

Representative ratemeters showing the spontaneous firing and the responses to a noxious stimulation of a single NS neuron following spinal application of PMF_2α_ (16 nmol) alone or following administration of AGN 211336 (6 nmol) in healthy rats, or the spontaneous firing and the responses to a noxious stimulation of a single NS neuron following spinal application of AGN 211336 (6 nmol) in inflamed knee rats, are shown in Fig. 6.

### Effect of PGF_2α_ alone or in combination with AL8810 or AGN211336 on the spontaneous activity of NS neurons

**Figure 7:**
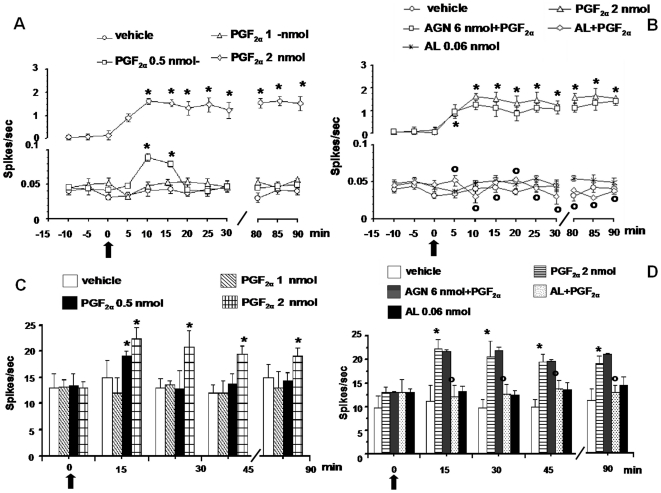
Effects of spinal application (microinjections) of vehicle (0.05% DMSO in ACSF), PGF_2α_ (0.5, 1 and 2 nmol) alone (A and C) or in combination with AGN 211336 (6 nmol) or AL 8810 (0.06 nmol) (B), and effects of AL 8810 (0.06 nmol) alone (B and D) on the spontaneous firing of NS neurons and on the evoked activity of NS neurons. Vehicle or drugs were administered at time 0, as indicated by the black arrow, whereas AGN 211336 (6 nmol) or AL 8810 (0.06 nmol) were administered 10 min before (not shown). Each point represents the mean ± S.E.M of 6–7 neurons of different treated group of mice. * indicates statistically significant difference versus vehicle, and ° versus PGF_2α_ (2 nmol). P values<0.05 were considered statistically significant (one-way ANOVA).

**Figure 8:**
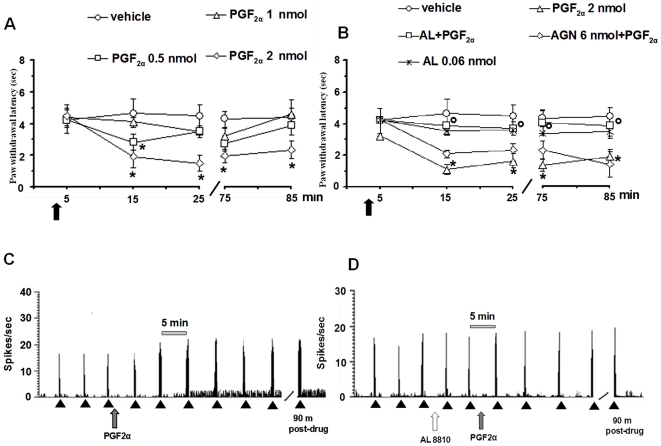
Effects of spinal application (microinjections) of vehicle (0.05% DMSO in ACSF), PGF_2α_ (0.5, 1 and 2 nmol) alone (A), or in combination with AGN 211336 (6 nmol) or AL 8810 (0.06 nmol) (B), and the effects of AL 8810 (0.06 nmol) alone (B), on mouse paw withdrawal latency (PWL). Vehicle or drugs were administered at time 0, as indicated by the black arrow, whereas AGN 211336 (6 nmol) or AL 8810 (0.06 nmol) were administered 10 min before (not shown). Each point represents the mean ± S.E.M 7 mice per group. * indicates statistically significant difference versus vehicle, and ° versus PGF_2α_ (2 nmol). P values<0.05 were considered statistically significant (one-way ANOVA). Representative ratemeters show the spontaneous activity and the responses to a noxious stimulation (von Frey filaments 97.8 mN/2 sec) of a single NS neuron both before and after spinal application of PGF_2α_ (2 nmol) alone, which increased the spontaneous activity and the noxious stimulation-evoked activity of NS neurons (C), or in combination with AL 8810 (0.06 nmol), which prevented the effect induced by PGF_2α_ (2 nmol) alone on NS spontaneous activity or on evoked activity (D). Scale grey bar indicates 5 min intervals for ratemeter records and small black arrows indicate the noxious stimulation on mouse hind paw.

Topical spinal cord application of vehicle (0.05% DMSO in ACSF) did not change the spontaneous activity of NS neurons (0.035±0.0032 spikes/sec) (Fig. 7A and 7B). Microinjections of PGF_2α_ (2 nmol) significantly increased the NS cell activity (1.52±0.15 spikes/sec; *P*<0.05), from 10 min from injection until the end of NS spontaneous activity recording (90 min) (Fig. 7A and 8C). The other doses of PGF_2α_ (0.5 and 1 nmol) did not change NS spontaneous activity. Pre-treatment with AGN211336 (6 nmol) did not prevent the effect of PGF_2α_ (2 nmol) on NS cell spontaneous activity (Fig. 7B), which was instead completely abolished by 10 min pre-treatment with a per se inactive dose of AL8810 (0.06 nmol) (Fig. 7B and 8D).

### Effect of PGF_2α_ alone or in combination with AL8810 or AGN211336 on evoked activity of NS neurons

Topical spinal cord application of vehicle (0.05% DMSO in ACSF) did not change the evoked activity of NS neurons (12.64±2.6 spikes/sec) (Fig. 7C and 7D). Microinjections of PGF_2α_ (2 nmol) significantly increased NS evoked activity (22.32±2.2 spikes/sec; *P*<0.05), from 15 min from injection until the end of NS evoked activity recording (90 min) (Fig. 7C). The other doses of PGF_2α_ (0.5 and 1 nmol) did not change NS evoked activity. Pre-treatment with AGN211336 (6 nmol) did not prevent the effect of PGF_2α_ (2 nmol) on NS evoked activity (Fig. 7D), which was instead completely abolished by 10 min pre-treatment with a per se inactive dose of AL 8810 (0.06 nmol) (Fig. 7D).

### Effect of PGF_2α_ alone or in combination with AL8810 or AGN211336 on the paw withdrawal latency (PWL)

Topical spinal cord application of vehicle (0.05% DMSO in ACSF) did not change the mouse paw PWL (4.5±1.8 sec) (Fig. 8A and 8B). Microinjections of PGF_2α_ (2 nmol) significantly reduced mouse PWL (1.8±2.2 spikes/sec; *P*<0.05), from 15 min from injection until the end of PWL evaluation (85 min) (Fig. 8A). The other doses of PGF_2α_ (0.5 and 1 nmol) did not change mice PWL. Pre-treatment with AGN211336 (6 nmol) did not prevent the effect of PGF_2α_ (2 nmol) on PWL (Fig. 8B), which was instead completely abolished by 10 min pre-treatment with AL8810 (0.06 nmol). AL8810 (0.06 nmol) was inactive *per se* (Fig. 8B).

## Discussion

The present study was aimed at evaluating the hypothesis that knee inflammation causes production of COX-2 metabolites of endocannabinoids in the spinal cord. This hypothesis was based on: a) previous findings indicating that COX-2 inhibitors can produce anti-hyperalgesic effects and reduce dorsal horn neuron firing in rodents with inflammatory pain in a manner attenuated by cannabinoid receptor antagonists; and b) the concept that, if spinal endocannabinoids reduce pain during inflammation and are degraded not only by FAAH and MAGL, but also by COX-2, inhibition of this enzyme should reduce endocannabinoid degradation and contribute to indirect activation of cannabinoid receptors [7], [14], whereas inhibition of FAAH or MAGL alone might not be sufficient to reduce inflammatory pain [6]. We report here for the first time that induction of knee inflammation in mice is accompanied by a strong elevation in the spinal cord of the COX-2 and prostaglandin F-synthase derivative of AEA, PMF_2α_, whereas other potential COX-2 endocannabinoid derivatives, such as PME_2_, PGF_2α_-GE and PGE_2_-GE were not detectable. At the same time, we observed that the spinal levels of AEA or 2-AG were not significantly elevated during knee inflammation, a finding that differs from that previously reported in a rat model of neuropathic pain [19]. In fact, the mean value corresponding to AEA levels decreased, albeit not significantly, by an amount exactly equivalent to the increase of PMF_2α_ (∼10 pmol/g), possibly in agreement with the hypothesis that the latter metabolite is produced from AEA. Finally, we showed that intrathecal PMF_2α_ exerts pro-algesic effects in the PWL test and enhances the firing of NS neurons in healthy mice in a way antagonised by a selective prostamide receptor antagonist but not by an FP receptor antagonist, and that the former antagonist attenuates hyperalgesia and NS neuron hyperxcitability in mice with knee inflammation. Taken together, these data indicate that in the model of inflammatory pain used here, possibly due to over-expression of COX-2 [20], AEA is converted to PMF_2α_, thus shifting the spinal cord from a CB_1_-mediated anti-hyperalgesic tone to a prostamide receptor-mediated pro-hyperalgesic tone, and contributing to NS neuron hyperexcitability and pain transmission. These findings are particularly timely in view of the very recent report that sensory neurons produce endocannabinoid COX-2 derivatives in vitro in a manner sensitive to (*R*)-flurbiprofen, which instead is a weak inhibitor of COX-2-mediated oxygenation of arachidonic acid and yet is endowed with anti-inflammatory and analgesic effects [13].

Interestingly, the production of PMF_2α_ was antagonised not only by a non-selective COX inhibitor and a selective COX-2 inhibitor, as expected, but also by a selective COX-1 inhibitor. This finding is in contrast with the well established notion that AEA and 2-AG are substrates of COX-2, but not COX-1 [17], [18]. In the previous report [7] that served as a starting point for the present study, the authors reported that the same non-selective COX, as well as selective COX-1 and COX-2, inhibitors used here, at intrathecal doses similar to those used here, were able to reduce pre-emptively the generation of inflammation-evoked NS neuron hyperexcitability and the formation of prostaglandin E_2_ in rats with knee inflammation. However, the effect of the COX-1 inhibitor was short-lasting and less efficacious than that of the COX-2 inhibitor, and only the latter still inhibited NS neuron hyperexcitability 7 h after the establishment of inflammation. Therefore, our findings, taken together with those of Telleria-Diaz and co-workers [7], seem to suggest that, in rodents with knee inflammation: 1) both spinal COX-1 and COX-2, through the formation of prostanoids, participate in the early events caused by inflammation and leading to NS neuron hyperexcitability and nociception; 2) the formation of spinal PMF_2α_ form AEA, catalysed selectively by COX-2, is, however, secondary to inflammation-induced pain and spinal hyperexcitability, and the reduction of PMF_2α_ levels by the COX-1 inhibitor reflects the effects of this substance on the above early events, rather than the direct inhibition of PMF_2α_ biosynthesis from AEA; 3) spinal PMF_2α_ contributes to maintain pain and NS neuron hyperexcitability, thus establishing a vicious circle, than can be interrupted by COX inhibition, and more effectively by COX-2 blockade. This hypothesis provides a further explanation as to why COX-2, but not COX-1, inhibitors are more effective at reducing NS neuron firing and capable of exerting this property also after the establishment of inflammation [7].

In their study on rats with knee inflammation, Telleria-Diaz and co-workers did not measure spinal AEA levels, but provided indirect evidence that COX-2 was responsible for the oxidation of 2-AG, since a selective COX-2 inhibitor, but not a non-selective COX inhibitor nor a selective COX-1 inhibitor, prevented the decrease of spinal 2-AG levels during inflammation. Accordingly, a CB_1_ receptor antagonist attenuated the effect of the COX-2 inhibitor on NS neuron hyperexcitability. However, the authors did not investigate the formation of spinal COX-2 derivatives of 2-AG (i.e. PG-GEs). In the present study in mice we did not detect any such derivative (even though we only investigated the presence of two major PG-GE species), nor could we see any decrease of spinal 2-AG levels following knee inflammation. This discrepancy between the two studies might be due species differences. However, it is also possible that we could not detect PG-GEs due to the sensitivity limits of our method, or that the formation of spinal PMF_2α_ in rats was simply left undetected in the previous study. At any rate, we did present here data strongly suggesting that COX-2-catalysed oxidation of endocannabinoids, and AEA in particular, is not only a way to inactivate endogenous mediators acting at anti-hyperalgesic cannabinoid receptors in the spinal cord, as suggested by previous authors [7], [14], but also a potential way to generate novel endogenous mediators acting at specific pro-algesic molecular targets different from prostanoid receptors.

In conclusion, we have reported here for the first time a sensitive and highly accurate method to measure COX-2 derivatives of endocannabinoids, and its application for the identification of one such compound, PMF_2α_, in the spinal cord of mice with knee inflammation. In fact, previous identification and quantification of prostamides by LC-MS-MS methods [11], [13] was not performed using a high resolution MS technique, such as the one employed here, which allows to establish with confidence the molecular formula of analytes, and was only carried out in vitro [12], [13], or in vivo in transgenic mice administered with exogenous AEA [11]. We have also described data suggesting for PMF_2α_ a role as a “late” mediator contributing to sustain spinal cord hyperexcitability and pain perception via activation of prostamide, by not FP, receptors. Thus, the present study provides novel and crucial data on a so far poorly investigated family of lipid mediators. Future studies will have to address the question of whether PMF_2α_, or other similar compounds, are produced in other models of inflammation, thus opening the way to the potential use of selective prostamide receptor antagonists as novel anti-hyperalgesic agents.

## Materials and Methods

### Ethics statement

The experimental procedures were approved by the Animal Ethics Committee of the Second University of Naples (decree nr. 98/2009-B). Animal care was in compliance with the IASP and European Community (E.C. L358/1 18/12/86) and with Italian (D.L. 116/92) guidelines on the use and protection of animals in experimental research. All efforts were made to minimise animal suffering and the number of animals used.

### Extraction of COX-2-derivatives of development of an LC-MS method for their quantification

Extraction, purification and quantification of AEA, 2-AG and PMF_2α_, PME_2_, PGF_2α_-GE and PGE_2_-GE from tissues require several biochemical steps. First, tissues were dounce-homogenized and extracted with acetone containing internal deuterated standards for AEA, 2-AG, PMF_2α_, PME_2_, PGF_2α_-GE and PGE_2_-GE quantification by isotope dilution ([^2^H]_8_AEA, [^2^H]_5_2AG, [^2^H]_4_ PMF_2α_, [^2^H]_4_ PME_2_, [^2^H]_4_ PGF_2α_ -GE and [^2^H]_4_ PGE_2_-GE). The lipid-containing organic phase was dried down, weighed and pre-purified by open bed chromatography on silica gel. Fractions were obtained by eluting the column with 99∶1, 90∶10, 70∶30 and 50∶50 (v/v) chloroform/methanol. The 90∶10 fraction was used for AEA and 2-AG quantification by liquid chromatography-atmospheric pressure chemical ionization-mass spectrometry (LC-APCI-MS), as previously described and using selected ion monitoring at M+1 values for the four compounds and their deuterated homologues, as described in [21]. The 70∶30 fraction was used for COX-2 derivatives quantification by LC-MS-IT-TOF analysis (Shimadzu Corporation, Kyoto, Japan) equipped with an ESI interface, using multiple reaction monitoring. The chromatograms of the high-resolution M+Na^+^ values were extracted and used for calibration and quantification. AEA and 2-AG were measured as previously described [21]. COX-2 derivatives were measured by LC-MS-MS, using an LC20AB coupled to a hybrid detector IT-TOF (Shimadzu Corporation, Kyoto, Japan) equipped with an ESI interface. LC analysis was performed in the isocratic mode using a Discovery® C18 column (15 cm×2.1 mm, 5 µm) and methanol/water/acetic acid (53∶47∶0.05 by vol.) as mobile phase with a flow rate of 0.15 ml/min. Identification of PME_2_, PMF_2α_, PGE_2_-GE and PGF_2α_-GE was carried out using ESI ionization in the positive mode with nebulizing gas flow of 1.5 ml/min and curved desolvation line temperature of 250°C.

For the representative experiment shown in Fig. 1, a rat brain homogenate was spiked with undeuterated PME_2_, PMF_2α_, PGE_2_-GE and PGF_2α_ -GE (100 pmol each) and processed as above.

### Drugs

PMF_2α_, PGF_2α_, PGF_2α_–GE, PGE_2_-GE, [^2^H]_4_ PMF_2α_, [^2^H]_4_ PME_2_, [^2^H]_4_ PGF_2α_-GE and [^2^H]_4_ PGE_2_-GE were provided by Allergan, CA, USA and and AGN211336 [9] by Selcia, UK. SC-560, NS-398, [^2^H]_8_AEA and [^2^H]_5_2AG were purchased from Cayman Chemicals (MI, USA); AL8810 [22], indomethacin, kaolin and λ-carrageenan were purchased from Sigma-Aldrich (Milano, Italy). All drugs were dissolved in 0.05% DMSO in ACSF.

### Animals

Male ICR (CD-1) mice (35–40 g) were housed 3 per cage under controlled illumination (12∶12 h light∶dark cycle; light on 06.00 h) and environmental conditions (room temperature 20–22°C, humidity 55–60%) for at least 1 week before the commencement of experiments. Mouse chow and tap water were available *ad libitum*.

### Inflammatory pain model

The induction of knee joint inflammation has been performed accordingly with Telleria-Diaz et al., 2010 [7]. Briefly, a 26-gauge needle was introduced through the patellar ligament of anesthetized mice (sodium pentobarbital, 60 mg/kg i.p.), and 40 µl of a 4% kaolin suspension were slowly injected into the articular cavity. After flexing and extending the joint slowly for 15 min, 40 µl of a 2% λ-carrageenan solution were injected, and the joint was moved for another 5 min.

### Electrophysiological recordings

On the day of electrophysiological recordings, mice were initially anesthetized with sodium pentobarbital (60 mg/kg i.p.). After tracheal cannulation, a catheter was placed into the right external jugular veins, to allow continuous infusion of propofol (5–10 mg/kg/h, i.v.) and spinal cord segments L4–L6 were exposed by laminectomy, medially near the dorsal root entry zone up to a depth of ∼1000 µm [23]. An elliptic rubber ring (about 3×5 mm) was tightly sealed with silicone gel onto the surface of the cord. This ring formed a trough with about 10–15 µl capacity over the spinal segments used for topical spinal drug application and to gain access to spinal neurons that receive input from either the ipsilateral paw, were the mechanical stimulation was applied, or knee where the inflammation was induced. Animals were then secured in a stereotaxic apparatus (David Kopf Instruments, Tujunga, CA, USA) supported by clamps attached to the vertebral processes on either side of the exposure site. The exposed area of the spinal cord was initially framed by agar and then filled with mineral oil. Body temperature was maintained at 37°C with a temperature-controlled heating pad [7], [23]. A glass-insulated tungsten filament electrode (3–5 MΩ) (FHC Frederick Haer & Co., ME, USA) was used to record single unit extracellular activity of dorsal horn NS neurons. NS neurons were defined as those neurons that respond only to high-intensity (noxious) stimulation [23]. For normal and inflamed knee animals, each neuron was characterized after mechanical stimulation of the ipsilateral hind paw by von Frey filament with bending force of 97.8 mN (noxious stimulation) for 2 s with it slightly buckled [24], [25], [26] to confirm NS response patterns. Only neurons that specifically responded to noxious hind paw stimulation, without responding to stimulation of the surrounding skin/tissue, were kept for recordings in normal and inflamed knee mice. The recorded signals were amplified and displayed on a digital storage oscilloscope to ensure that the unit under study was unambiguously discriminated throughout the experiment. Signals were also fed into a window discriminator, whose output was processed by an interface CED 1401 (Cambridge Electronic Design Ltd., UK) connected to a Pentium III PC. Spike2 software (CED, version 4) was used to create peristimulus rate histograms on-line and to store and analyse digital records of single unit activity off-line. Configuration, shape, and height of the recorded action potentials were monitored and recorded continuously using a window discriminator and Spike2 software for on-line and off-line analysis. This study only included neurons whose spike configuration remained constant and could clearly be discriminated from activity in the background throughout the experiment, indicating that the activity from one neuron only and from the same one neuron was measured. In each mouse, only one neuron was recorded before and after vehicle or drug administration. After characterization, three baseline responses, separated by 5 min each, to specific stimulation (see below) of the NS neurons were recorded. Spontaneous and evoked neuronal activity was then measured, after spinal vehicle or drug applications, in the 5 minutes leading up to each stimulus up to 90 min and up to 120 min in normal knee and inflamed knee mice, respectively, and was expressed as spikes/sec (Hz). A mean of the three pre-drug spontaneous and evoked activity was calculated to represent baseline evoked activity. For each neuron, the post-drug spontaneous and evoked activity was calculated as a percent of the respective baseline levels. At the end of the experiment, each animal was killed with a lethal dose of urethane.

### Behavioural tests

After surgical preparation as previous described for electrophysiological experiments, thermal hyperalgesia was evaluated by using a thermal stimulus elicited by a radiant heat source as well as the tail flick unit (Ugo Basile, Varese, Italy) focused on the mouse plantar surface of the hind paw. The paw was placed over the surface of a slightly projecting window receiving the I.R. energy. The I.R. intensity in our experiments has been set to 50 C° [27]. Nociceptive responses for thermal sensitivity were expressed as thermal PWL in seconds and it was determined by a timer connected to a photoelectric cell which stopped the timer (and switched off the lamp) at the movement of the paw which was withdrawn. PWL was misured every 5 min for at least 15 min prior to microinjecting drugs, or the respective vehicle, 0.05% dimethyl sulfoxide (DMSO) in artificial cerebrospinal fluid (ACSF, composition in mM: KCl 2.5; NaCl 125; MgCl_2_ 1.18; CaCl_2_ 1.26), on spinal cord surface.

### Treatments

Animals receiving spinal (intrathecal) application of 2 µl vehicle (DMSO/ACSF, 0.05%, v/v) or drug solutions were grouped as follows:

1. Normal knee mice treated with: a) vehicle; b) PMF_2α_ (4, 8 and 16 nmol); c) PMF_2α_ (16 nmol) in combination with AGN211336 (6 nmol) or AL8810 (0.06 nmol), a prostamide antagonist and a selective FP prostanoid receptor antagonist, respectively; d) AGN 211336 (6 nmol) or AL 8810 (0.06 nmol) alone; e) PGF_2α_ (0.5, 1 and 2 nmol); f) PGF_2α_ (2 nmol) in combination with AGN211336 (6 nmol) or in combination with AL8810 (0.06 nmol);
2. Sham knee mice (receiving only saline in the knee) treated with vehicle, for behavioural studies and electrophysiological recordings;
3. Inflamed knee mice treated with: a) vehicle; b) AGN 211336 (6 nmol), for behavioural studies and electrophysiological recordings.

For nociceptive behaviour and in vivo single-unit extracellular recordings, experimental groups consisted of 7 mice. Only one neuron was recorded in each mouse. When AGN211336 and/or AL8810 were used in combination with PMF_2α_ or PGF_2α_, the latter compounds were administered 10 minutes after the antagonists. Drug doses were chosen according to previous experiments [9], [28], [29].

Finally, groups of 4 healthy mice and/or of mice knee inflammation were treated with: a) vehicle; b) indomethacin, a non-selective COX inhibitor (8 mM); c), SC-560, a selective COX-1 inhibitor (3 mM); and d) NS-398, a selective COX-2 inhibitor (1.3 mM). The volume injected was always 5 µl. COX inhibitors were intratecally administered 7 h after induction of acute kaolin/λ-carrageenan inflammation. One hour post COX inhibitor injections mice were sacrificed and whole spinal cord (L4–L6) explanted and used for the quantification of endocannabinoids and their COX-2 derivatives. Drugs doses were chosen accordingly with previous experiments [7], [30].

### Statistics

Behavioural and electrophysiology data are represented as means ± SEM and statistical analysis of these data were performed by two way ANOVA for repeated measured followed by the Student-Newman-Keuls for multiple comparisons to determine statistical significance between different treated groups of mice.
